# Pancreaticobiliary Maljunction Is Associated with Common Bile Duct Carcinoma: A Meta-Analysis

**DOI:** 10.1155/2013/618670

**Published:** 2013-12-30

**Authors:** Yang Li, Jun Wei, Zhongxin Zhao, Tiangeng You, Mingan Zhong

**Affiliations:** Department of Gastrointestinal Surgery, East Hospital, Tongji University, 150 Jimo Road, Pudong New District, Shanghai 200120, China

## Abstract

*Objective*. Pancreaticobiliary maljunction (PBM) has been reported to be associated with an increased risk of gallbladder carcinoma. However, the relationship between PBM and common bile duct carcinoma (CBDC) remains unclear. We aimed to conduct a meta-analysis to determine the available evidence on the association between PBM and CBDC. *Methods*. The pooled odds ratio (OR) and standard mean differences (SMD) with 95% confidence interval (95% CI) were used to estimate the effects. *Results*. A total of eight case-control studies and two cohort studies were identified. The incidence of PBM was higher in CBDC patients than in controls (OR = 1.45; 95% CI, 1.19–1.76). Compared with patients without PBM, CBDC patients with PBM were younger at the diagnosis age (SMD = −0.46; 95% CI, −0.64 to −0.28). A difference in the incidence of associated CDC was found between CBDC patients with or without PBM (OR = 2.14; 95% CI, 1.60–2.87). *Conclusions*. Compared with benign biliary tract diseases, the incidence of PBM was higher in CBDC patients, especially in relatively young patients. We also found that the incidence of CDC was higher in CBDC patients with PBM. These findings showed positive association between PBM and CBDC, which may help in identifying high-risk individuals.

## 1. Introduction

Common bile duct carcinoma (CBDC) is a malignancy arising from the ductular epithelium of the biliary tree, mainly located in the distal common bile duct, which accounts for 20–30% of all bile duct carcinomas (BDC) [1]. CBDC accounts for 1% of all gastrointestinal cancers [2]. But, it is more prevalent in Eastern Asian countries, including China [1, 3, 4]. The peak age for patients with CBDC is the seventh decade and the sex incidence shows a slight male preponderance [5, 6]. The five-year survival rate of CBDC patients following surgical resection is approximately 5–15% [6, 7]. These figures represent the aggregate prognosis of all patients with CBDC [5–7]. Therefore, it is important to identify the high-risk group for CBDC, especially in high-incidence countries, such as Japan, Korea, China, and Pakistan.

It is widely known that pancreaticobiliary maljunction (PBM) is an anomalous arrangement of pancreaticobiliary ductal system with the union of the pancreatic and biliary ducts locating outside the duodenal wall [8–11]. As the action of the sphincter muscle does not functionally affect the union, two-way regurgitation (pancreaticobiliary and biliopancreatic reflux) occurs, resulting in various pathological conditions in the biliary tract and pancreas [9, 12–15]. Hasumi et al. [16] revealed that the incidence of PBM was 3.3% among 12,399 patients who underwent hepatobiliary tract surgery and approximately 4.4% of the common bile duct cancer patients had PBM. Therefore, the positive correlation between PBM and CBDC has drawn increasing attention [17, 18].

Kamisawa et al. [12] collaborating with Working Committee of Clinical Practice Guidelines for Pancreaticobiliary Maljunction divided PBM into three distinct types based on imaging findings: (1) bile duct (junction) type, in which the bile duct joins the pancreatic duct at a right angle; (2) pancreatic duct (junction) type, in which the pancreatic duct joins the bile duct at an acute angle; (3) complex type, in which the two ducts meet in such a complex manner that the junction cannot be classified as either of the previous two types.

To elucidate the effect of PBM on the development of CBDC, we thus carried out a comprehensive meta-analysis of the current epidemiological literature to investigate the associations between the two diseases.

## 2. Methods

### 2.1. Literature Sources and Searches

Bibliography search was carried out in PubMed (1970 to December 2012) and Web of Science (1986 to December 2012). All aspects of the Preferred Reporting Items for Systematic Reviews and Meta-Analysis (PRISMA) statement were followed [19]. The medical subject heading (MeSH) terms and key words used in the search included “pancreaticobiliary maljunction,” “anomalous pancreaticobiliary ductal junction,” “anomalous pancreaticobiliary ductal union” combined with “bile duct carcinoma(s),” or “bile duct cancer(s),” or “bile duct tumor(s),” or “bile duct neoplasm(s),” or “bile duct malignancy(ies).”

### 2.2. Study Selection

We included studies that met the following criteria: (i) case-control and cohort studies published in English; (ii) PBM as one of the exposures of interest and CBDC as one of the outcomes of interest (iii) conformation of PBM by endoscopic retrograde cholangiopancreatography (ERCP) or percutaneous transhepatic cholangiography (PTC) or magnetic resonance cholangiopancreatography (MRCP); (iv) diagnosis of CBDC based on surgery or pathology; and (v) reporting one of the following clinical data in patients with CBDC combined with PBM: age, sex, associated CDC, and type of PBM.

### 2.3. Data Extraction

The parameters from studies were extracted and entered into a database. The following data were collected: first author, year of publication, study design, country of origin, number of participants (cases and controls), potential confounders, diagnostic methods for PBM and bile duct carcinoma, and clinical data on patients with bile duct carcinoma associated with PBM, OR, and 95% confidence interval (CI). The authors were contacted for additional information, when necessary. Data were independently extracted and analyzed by two researchers (Yang Li and Zhongxin Zhao) and final decision was reached by consensus, referring back to the original article.

### 2.4. Statistical Analysis

Summary OR estimates with their corresponding 95% CI were calculated with a fixed-effects or random-effects model. Heterogeneity across studies was tested with the *Q* and *I* ^2^ statistics. For the *Q* statistic, the results were defined heterogeneous for *P* < 0.10. A random-effects model was applied in the occurrence of significant heterogeneity (*P* < 0.10). For *I* ^2^, a value of more than 50% was considered a measure of severe heterogeneity [20]. We conducted analyses stratified by study design, diagnosis age, and the incidence of CDC. Publication bias was assessed by Begg's funnel plot and Egger's test; the former was based on adjusted rank correlation and the latter on a regression model (*P* < 0.10 as an indication for publication bias) [21, 22]. All statistical analyses were performed with STATA, version 11.0 (STATA, Corp., College Station, Lakeway Drive, TX, USA).

## 3. Results

### 3.1. Search Results

**Figure 1:**
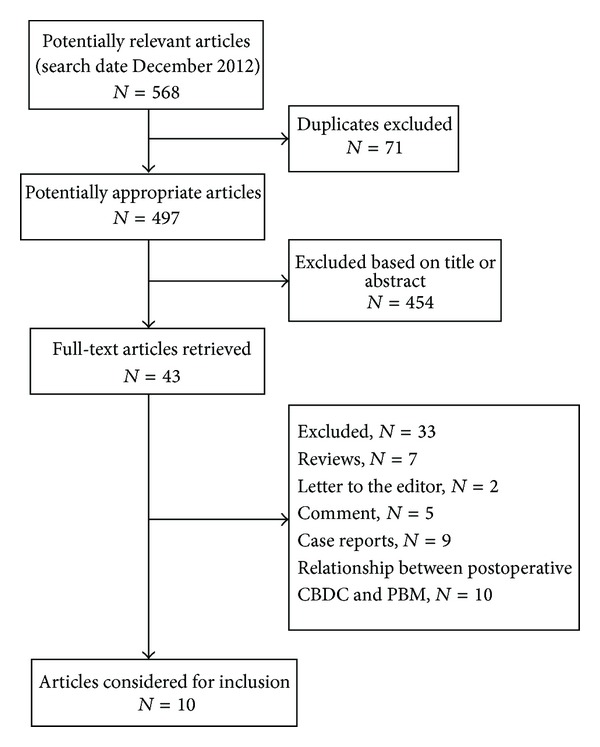
Article search flow chart showing the inclusion and exclusion of studies.

568 articles were identified and screened by querying PubMed and Web of Science through December 2012, of which 71 were duplicates (Figure 1). Most of them (*n* = 454) were not focused on the relation between PBM and common bile duct carcinoma and were therefore not considered. Of the remaining 43 articles, 33 did not meet the inclusion criteria. Thus, the present analyses were based on 8 case-control studies and 2 cohort studies. A total of 2139 incident CBDC cases (144 incident PBM and 1995 incident without PBM) and 23,967 incident nonmalignant pancreaticobiliary diseases were included in this meta-analysis.

### 3.2. Characteristics of Eligible Studies

The characteristics of these studies were shown in Table 1. Ten clinical trials [8, 10, 13, 16, 18, 23–27] were identified to give the information with the incidence of PBM in CBDC patients and controls according to the inclusion criteria in the meta-analysis. After the communication with authors of the above studies, some information remains unclear with objective reasons. Wang et al. [23] and Suda et al. [26] did not provide the diagnosis age of CBDC patients with or without PBM. Suda et al. [26] did not provide the incidence of CDC in CBDC patients with PBM.

### 3.3. Meta-Analysis Results

**Figure 2:**
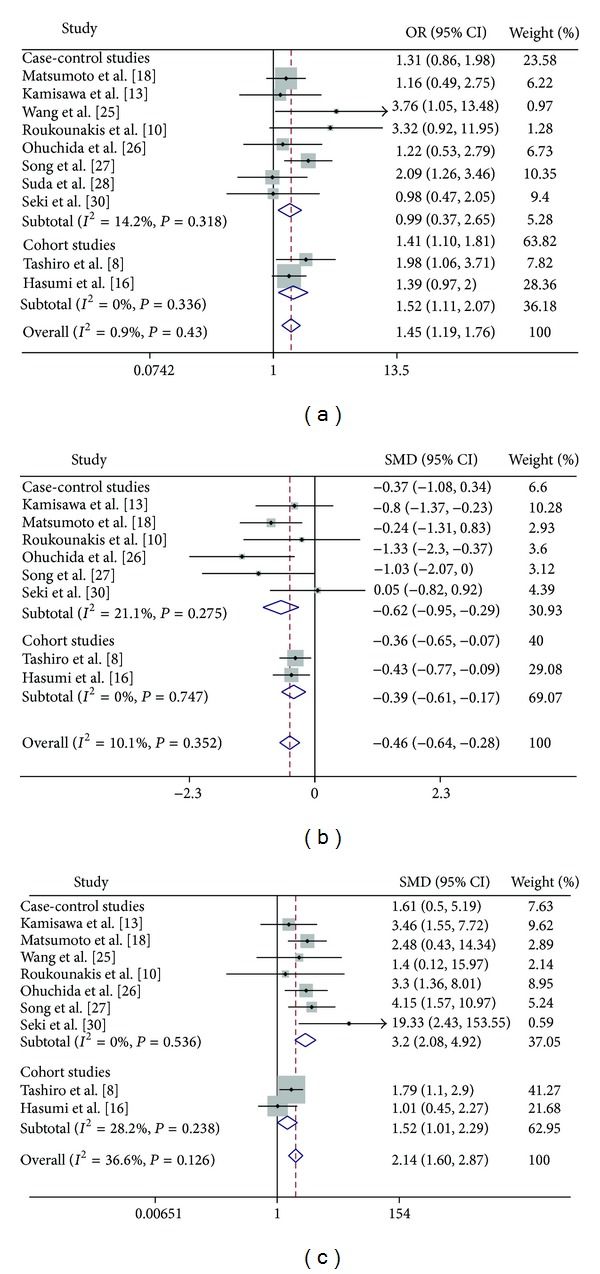
Forest plot of the association between PBM and CBDC risk. (a) Summary risk of CBDC associated with PBM. (b) Forest plot describing the SMD for diagnosis age in CBDC patients with or without PBM. (c) Forest plot describing the OR of the incidence of CDC in CBDC patients with or without PBM. PBM: pancreaticobiliary maljunction; CBDC: common bile duct carcinoma; SMD: standard mean difference; CI: confidence interval; OR: odds ratio.

The OR estimates for the association between PBM and CBDC are shown in Figure 2(a). The OR estimates from eight case-control studies (OR = 1.41, 95% CI: 1.10–1.81), two cohort studies (OR = 1.52, 95% CI: 1.11–2.07), and all studies combined (OR = 1.45, 95% CI: 1.19–1.76) were statistically significant and showed a positive association with PBM. The pooled incidence of PBM was 6.73% (144 of 2139) in CBDC patients and 5.10% (1223 of 23967) in controls. The fixed-effects model was used because the test for heterogeneity was not statistically significant (*P* = 0.43, *I* ^2^ = 0.9%).

Diagnosis age in each study was extracted as mean ± SD (Table 2). The Forest plot for mean age at diagnosis in CBDC patients with or without PBM was displayed in Figure 2(b). There is no substantial heterogeneity (*P* = 0.35, *I* ^2^ = 10.1%) and the fixed-effects model was used. The OR were −0.62 (95% CI: −0.95 to −0.29) from the six case-control studies, −0.39 (95% CI: −0.61 to −0.17) from the two cohort studies, and −0.46 (95% CI: −0.64 to −0.28) overall, which revealed that the mean age at diagnosis for CBDC in PBM patients was younger than that of patients without PBM.

Overall there was no heterogeneity amongst the 9 studies (seven case-control studies and 2 cohort studies) when examining the incidence of CDC in CBDC patients with or without PBM (*P* = 0.13, *I* ^2^ = 36.6%) and the fixed-effects model was used (Table 2). The Forest plot was shown in Figure 2(c): the OR estimate from the seven case-control studies was 3.20 (95% CI: 2.08–4.92) and from the two cohort studies was 1.52 (95% CI: 1.01–2.29) and the overall estimate was 2.14 (95% CI: 1.60–2.87). The results suggest that there is a positive correlation between the increased risk of CBDC and the incidence of CDC with PBM.

### 3.4. Publication Bias

**Figure 3:**
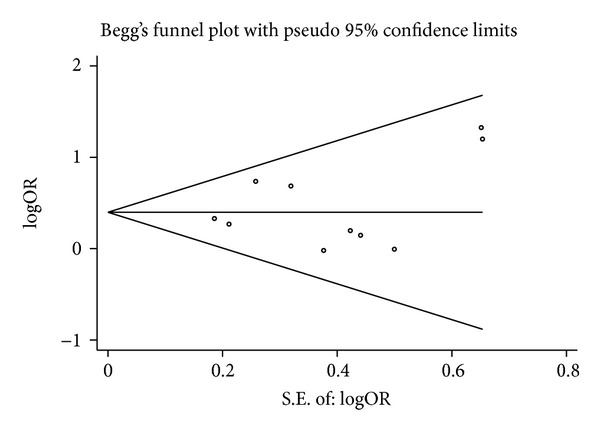
Funnel plot of studies evaluating the relationships between PBM and risk of CBDC. PBM: pancreaticobiliary maljunction; CBDC: common bile duct carcinoma; S.E.: standard error; OR: odds ratio.

Figure 3 showed that no evidence of publication bias was found from both visualization of Begg's funnel plot and Egger's test. *P* values for Begg's adjusted rank correlation test and Egger's regression asymmetry test were 0.858 and 0.506 for the association of PBM and CBDC risk, respectively.

## 4. Discussion

The results of this meta-analysis based on 10 studies (8 case-control studies and 2 cohort studies) provide supportive evidence of a positive association between PBM and CBDC. We could not detect any important difference between case-control and cohort studies, which provides support to the positive association observed.

PBM is an important carcinogenic factor in biliary tract carcinoma, especially in Asian patients. Many researches have been published to reveal a potential association between PBM and the development of CBDC; however, the association remains controversial. Tashiro et al. [8] and Tanaka et al. [28] reported that the number of bile duct cancers exceeded the number of gallbladder cancers in the dilated bile duct cases of PBM, while gallbladder cancer frequently occurred but bile duct carcinoma rarely occurred in PBM cases not associated with dilatation. Whereas Seki et al. [27] demonstrated that bile duct cancer did not occur in cystic dilatation, 50% of bile duct cancers developed from nondilated bile duct epithelium with PBM over 50 years old. The results from our meta-analysis indicate that patients with PBM have an increased risk of developing CBDC compared with those without PBM, especially those PBM patients with CDC.

One recent published meta-analysis has assessed PBM and the risk of developing gallbladder carcinoma. Deng et al. [15] combined six studies to investigate the difference in the incidence of PBM between gallbladder cancer patients and controls. The results indicated that the incidence of PBM among gallbladder cancer patients is significantly higher than that in general population. The combined OR was 7.41 (95% CI: 5.03–10.77). However, this study did not demonstrate the relationship between PBM and CBDC. As the CBDC is the common carcinoma associated with PBM, we consider that it is beneficial to evaluate the correlation between PBM and CBDC in a comprehensive analysis.

Several mechanisms have been proposed to potentially underlie the development of CBDC in individuals with PBM. The carcinogenesis of CBDC coexisting with PBM is considered to involve the hyperplasia-dysplasia-carcinoma sequence provoked by the chronic inflammation that is a result of the reflux of pancreatic juices into the biliary tract [11, 29]. Strong cytotoxic substances (such as lysolecithin, secondary bile acids, and deconjugated bile acids) are produced when phospholipase A2 in the pancreatic juice mixes with bile, which are recognized clinically and experimentally to be injurious to cell membranes, and as a result, the chronic inflammation provokes high proliferative activity in the mucosal epithelia, finally causing CBDC in PBM patients [30–32]. The carcinogenesis of common bile duct epithelial cells in PBM patients is a multigene pathological process, associated with many genetic mutations, such as K-*ras* and *p*53 [33–35]. In addition, pancreatic juice can easily flow into the common bile duct in C-P type in which the common bile duct joins the pancreatic duct at approximately 90°. This mechanism is thought to be different from the adenoma-carcinoma sequence or the de novo carcinogenesis associated with biliary tract cancer in the population without PBM [3].

PBM can provide stagnant sites exposed to a mixture of bile and pancreatic juice over a prolonged period. Prolonged exposure causes persistent chronic inflammation in the biliary lining epithelium, leading to hyperplasia, atypia, and ultimately carcinoma [36]. This suggests that the stagnant site is an indispensable factor for carcinogenesis. Such sites could be provided by the dilated cyst in patients with PBM accompanied by CDC, and canceration is more likely to occur within a dilated cyst. Interestingly, Kosaka et al. [37] reported that it is extremely common for *H. bilis* to colonize the biliary system in patients with PBM. Pathophysiological alterations, such as erosion, desquamation, hyperplasia, and gastric or intestinal metaplasia [17], along with bile stasis, may allow *H. bilis* to colonize the biliary system in PBM. *H. bilis* may play an important role in inflammation-associated biliary carcinogenesis.

The present meta-analysis must be interpreted in the context of several limitations: (1) 8 of 10 studies included in this meta-analysis used a case-control design, which is more susceptible to recall and selection biases than a cohort design; the other 2 cohort studies may be affected by detection bias as patients with PBM are under increased medical examination because of clinical symptoms such as abdominal pain, vomiting, jaundice, and fever and thus may be more likely to be diagnosed with CBDC. These biases may confuse the true association between PBM and CBDC. (2) The definition of PBM with the length of common channel varied across studies (>8 mm, 10 mm, or 15 mm) and this restricts the comparability of these studies. Therefore, some degree of inappropriate diagnosis of PBM is likely to occur. These nonuniform diagnostic criteria would tend to reduce the magnitude of the results. (3) Some subgroup analyses were based on few studies and the results need to be cautiously interpreted. Moreover, confounding is also likely to be present. Although most studies controlled for some confounding factors, such as smoking, and alcohol abuse, which have generally been associated with an increased risk of CBDC, we cannot exclude the possibility of residual confounding. (4) Other potential limitations of our study could be due to the language restriction in that we only selected articles published in English. (5) Finally, as in any meta-analysis, the possibility of publication bias is of concern, because small studies with null results tend not to be published. However, the results obtained from funnel plot analysis and formal statistical tests did not provide evidence for such bias.

In summary, this meta-analysis suggests an association between PBM and the risk of CBDC, especially for relatively young patients with CDC. However, to strengthen our findings, well-designed prospective studies with accurate diagnostic criteria of PBM may help to explore the relation between PBM and the risk of CBDC.

## Figures and Tables

**Table 1:** Characteristics of 10 studies included in this meta-analysis.

First author (year)	Country of origin	Study period	No. of cases	No. of controls	Diagnosis of PBM	Adjusted OR	95% CI
Case-control studies							
Kamisawa et al. [13] (2010)	Japan	1973–2009	107 cases; Asian	159 patients without CBDC; Asian	ERCP or PTC Common channel >15 mm in length and a lack of effect of the sphincter of Oddi at the union of the pancreatic and biliary ducts	1.16	0.49–2.75

Matsumoto et al. [18] (2003)	Japan	1983–2003	250 cases; Asian	2839 cases without CBDC; Asian	ERCP Common channel ≥10 mm in length or <10 mm and a lack of contraction of the Oddi at the union of the pancreatic and biliary ducts	1.31	0.86–1.98

Wang et al. [23] (1998)	Taiwan	1992–1996	27 cases; Asian	653 cases; Asian	ERCP Common channel ≥12 mm in length and/or one duct joining perpendicularly to another	3.76	1.05–13.48

Roukounakis et al. [10] (2007)	USA	1989–1998	26 cases; Caucasian	154 cases with normal films or nonmalignant pancreaticobiliary diseases for the same period; Caucasian	Cholangiopancreatogram and ERCP Common channel ≥8 mm in length or/and one duct joining perpendicularly to another	3.32	0.92–11.95

Ohuchida et al. [24] (2006)	Japan	1979–2004	196 cases; Asian	157 benign biliary diseases cases; Asian	ERCP and/or MRCP Common channel ≥15 mm in length on cholangiopancreatogram and a high level of amylase in bile	1.22	0.53–2.79

Song et al. [25] (1999)	Korea	1990–1996	329 cases; Asian	5037 cases underwent ERCP with benign biliary disease; Asian	ERCP The anomalous union of pancreaticobiliary duct system at a distance >15 mm from the papilla of Vater	2.09	1.26–3.46

Suda et al. [26] (1983)	Japan	1978–1983	72 cases; Asian	171 cases randomly selected and autopsied; Asian	Surgery	0.98	0.47–2.05

Seki et al. [27] (2005)	Japan	1988–2003	38 cases; Asian	145 cases without CBDC; Asian	ERCP or MRCP Common channel ≥10 mm and an anomalous junction of the pancreatic and biliary ducts located outside the duodenal wall	0.99	0.37–2.65

Cohort studies							
Tashiro et al. [8] (2003)	Japan	1990–1999	680 cases from the 141 hospitals throughout the country; Asian	2667 cases without biliary tract carcinoma; Asian	ERCP or PTC Common channel ≥15 mm in length and the junction located outside duodenal wall	1.98	1.06–3.71

Hasumi et al. [16] (2000)	Japan	1980–1984	414 cases from 133 institutions; Asian	11,985 benign biliary cases underwent hepatobiliary tract surgery at the same 133 institutions; Asian	ERCP or PTC Common channel ≥15 mm in length and union free of effect of sphincter	1.39	0.97–2.00

PBM: pancreaticobiliary maljunction; CBDC: common bile duct carcinoma; CDC: congenital dilatation of the common bile duct; ERCP: endoscopic retrograde cholangiopancreatography; MRCP: magnetic resonance cholangiopancreatography; PTC: percutaneous transhepatic cholangiography.

**Table 2:** The clinical data of included studies on CBDC patients with or without associated PBM.

First author (year)	With PBM			Without PBM		
	Number of patients	Age at diagnosis	Incidence of CDC	Number of patients	Age at diagnosis	Incidence of CDC
Tashiro et al. [8] (2003)	153	24.3 ± 23.9	29/153	527	47.3 ± 19.3	61/527
Kamisawa et al. [13] (2010)	16	54.6 ± 14.2	5/16	91	58.8 ± 9.1	20/91
Matsumoto et al. [18] (2003)	39	63.0 ± 11.0	12/39	211	71.4 ± 10.4	24/211
Wang et al. [23] (1998)	11	NA	4/11	16	NA	3/16
Roukounakis et al. [10] (2007)	22	65.6 ± 9.8	7/22	4	68.0 ± 11.5	1/4
Ohuchida et al. [24] (2006)	55	30.0 ± 20.7	12/55	141	52.9 ± 16.8	11/141
Hasumi et al. [16] (2000)	63	53.2 ± 11.9	8/63	351	66.5 ± 18.4	44/351
Suda et al. [26] (1983)	27	NA	NA	45	NA	NA
Song et al. [25] (1999)	38	36.5 ± 14.1	7/38	291	49.2 ± 12.2	15/291
Seki et al. [27] (2005)	6	51.6 ± 13.3	4/6	32	50.9 ± 13.7	3/32

PBM: pancreaticobiliary maljunction; CBDC: common bile duct carcinoma; CDC: congenital dilatation of the common bile duct; NA: not available.
